# Macrophages-derived exosomal lncRNA LIFR-AS1 promotes osteosarcoma cell progression via miR-29a/NFIA axis

**DOI:** 10.1186/s12935-021-01893-0

**Published:** 2021-04-01

**Authors:** Hongliang Zhang, Yiyang Yu, Jun Wang, Yu Han, Tingting Ren, Yi Huang, Chenglong Chen, Qingshan Huang, Wei Wang, Jianfang Niu, Jingbing Lou, Wei Guo

**Affiliations:** 1 Musculoskeletal Tumor Center, Peking University People’s Hospital, No. 11 Xizhimen South Street, Beijing, 100044 People’s Republic of China; 2 Beijing Key Laboratory of Musculoskeletal Tumor, Beijing, People’s Republic of China

**Keywords:** Bone tumor, Tumor-associated macrophages, Exosomal lncRNA, LIFR-AS1, Exosome

## Abstract

**Background:**

Osteosarcoma (OS) is the most common primary malignant bone tumor in young people. Tumor-associated macrophages (TAMs) have been reported to play an important role in the development of osteosarcoma. However, the detailed molecular mechanisms remain largely unknown and need to be elucidated. Recently, exosomes have been reported as the crucial mediator between tumor cells and the tumor microenvironment. And a lot of lncRNAs have been reported to act as either oncogenes or tumor suppressors in osteosarcoma. In this research, we aim to explore the role of macrophages-derived exosomal lncRNA in osteosarcoma development and further elucidated the potential molecular mechanisms involved.

**Methods:**

TAMs were differentiated from human mononuclear cells THP-1, and a high-throughput microarray assay was used to analyze the dysregulated lncRNAs and miRNAs in osteosarcoma cells co-cultured with macrophages-derived exosomes. Western blot, qRT-PCR assays, and Dual-luciferase reporter assay were used to verify the interaction among LIFR-AS1, miR-29a, and NFIA. Cck-8, EdU, colony formation assay, wound-healing, and transwell assay were performed to explore the characterize the proliferation and metastasis ability of OS cells. And qPCR, Western blots, immunohistochemistry, and cell immunofluorescence were used to detect the expression of relative genes or proteins.

**Results:**

In this study, we found that THP-1-induced macrophage-derived exosomes could facilitate osteosarcoma cell progression both in vitro and in vivo. Then, the results of the high-throughput microarray assay showed that LIFR-AS1 was highly expressed and miR-29a was lowly expressed. Furthermore, LIFR-AS1 was identified as a miR-29a sponge, and NFIA was validated as a direct target of miR-29a. Functional assays demonstrated that knockdown of exosomal LIFR-AS1 could attenuate the promotion effects of macrophages-derived exosomes on osteosarcoma cell progression and miR-29a inhibition could reserve the effect of LIFR-AS1-knockdown exosomes. Correspondingly, NFIA-knockdown could partially reverse the tumor inhibition effect of miR-29a on osteosarcoma cells.

**Conclusions:**

Taken together, macrophages-derived exosomal lncRNA LIFR-AS1 can promote osteosarcoma cell proliferation, invasion, and restrain cell apoptosis via miR-29a/NFIA axis, which can act as a potential novel therapeutic target for osteosarcoma therapy.

**Supplementary Information:**

The online version contains supplementary material available at 10.1186/s12935-021-01893-0.

## Background

Osteosarcoma is the most common primary malignant bone tumor [1]. It is believed to arise from malignant mesenchymal cells and the pathological process is extremely complex [2–4]. Although conventional treatment options such as chemotherapy and surgery are constantly improving, and new therapies such as targeted therapy and immunotherapy are also appearing, the prognosis of osteosarcoma patients is still poor due to the high degree of malignancy, rapid progression, and chemo-resistance of the disease [5, 6]. Therefore, it is urgent to elucidate the molecular mechanisms and explore the novel therapeutic strategy of osteosarcoma.

The communication between tumor cells and the corresponding microenvironment is critical for tumor growth and metastasis, and TAMs are the most abundant infiltrating immune cells and play critical roles in the progression of various tumors, including osteosarcoma [7–9]. Activated TAMs can be either antineoplastic (M1) or tumor-promoting (M2), and they often coexist at a dynamic change in a variety of tumors [10]. And our previous study had proved that TAMs could promote lung metastasis and induce epithelial-mesenchymal transition in osteosarcoma by activating the COX-2/STAT3 axis [11]. However, the communication between tumor cells and TAMs is still unclear.

Recently, more and more studies have reported that nano-sized exosomes can be crucial mediators between cellular communication, and cargos like long noncoding RNAs (lncRNAs) could be transported between cells [12, 13]. lncRNA is a type of noncoding RNA that participated in various biological processes in a wide range of human tumors, and a lot of lncRNAs have been reported to act as either oncogenes or tumor suppressors in osteosarcoma [14–16]. Leukemia inhibitory factor receptor antisense RNA 1 (LIFR-AS1) is a novel tumor-related lncRNA and has been reported as a tumor suppressor in breast cancer [17]. However, the role of LIFR-AS1 in osteosarcoma remains unknown.

In this research, we aim to explore the role of macrophages-derived exosomal lncRNA LIFR-AS1 in osteosarcoma development and further elucidated the potential molecular mechanisms involved.

## Methods and materials

### Clinical tissue specimen and microarray analysis

Osteosarcoma and normal bone tissues were acquired from the Musculoskeletal Tumor Center, Peking University People’s Hospital (Beijing, China). Informed consent was obtained from all patients and the research was approved by the Ethics Committee of Peking University People's Hospital.

### Cell culture and transfection

The human osteosarcoma cell lines 143B, HOS, MG63, MNNG, Saos2, and U2OS and the human monocytic cell line THP-1 cells were obtained from the American Type Culture Collection (ATCC, Manassas, VA, USA). The 143B, MNNG, and Saos2 cells were cultured in Dulbecco's modified Eagle's medium (DMEM, HyClone) supplemented with 10% fetal bovine serum (FBS, Gibco), and the HOS, KHOS, U2OS, and MG63 were maintained in RPMI-1640 medium (HyClone) with 10% FBS. All cells were cultured in a humidified incubator with 5% CO2 at 37 °C. Monocyte-differentiated macrophages were induced from THP-1 by treating with 100 ng/ml PMA for 24 h. Blood samples were donated by healthy volunteers, and peripheral blood mononuclear cells (PBMCs) were isolated using the Human Buffy Coat CD14 Positive Selection Kit (Stemcell) following the manufacturer's protocol.

Besides, si-LIFR-AS1, siNFIA, miR-29a mimic, miR-29a inhibitor, and corresponding negative control (NC) were obtained from Gene Pharm (Suzhou, China) and transfected by Lipofectamine 3000 (Invitrogen) according to the instructions provided.

### RNA isolation and qRT-PCR

Total RNA was isolated using TRIzol (Invitrogen) and miRNA was isolated by RNeasy/miRNeasy Mini Kit (Qiagen) according to the manufacturer’s instructions. U6 was used as the endogenous control of miRNAs and β-actin was used as the internal reference of other mRNAs.). The primers used in the experiments are listed in Table 1.

**Table 1:** Primers for real-time PCR

Primers		Sequences (5′–3′)
LIFR-AS1	F	GCAAATACTGTGTATTAGTCC
	R	CCGCTTCCTTGTGAAGAAGGT
NFIA	F	TAATCCAGGGCTCTGTGTCC
	R	CCTGCAGCTATTGGTGTCTG
β‐actin	F	GTCAGGTCATCACTATCGGCAAT
	R	AGAGGTCTTTACGGATGTCAACGT
miR-29a	F	5′-GGGTAGCACCATCTGAAAT-3’
	R	CAGTGCGTGTCGTGGAGT
U6	F	CTCGCTTCGGCAGCACA
	R	AACGCTTCACGAATTTGCGT

### Western blot

The total protein was isolated using cell lysis buffer and the procedure of western blot was the same as previously described [18]. The following antibodies, anti-cleaved-PARP, anti-E-cadherin, anti-N-cadherin were obtained from Cell Signaling Technology, anti-Bcl-2, anti-Bax, anti-NFIA were obtained from Proteintech, and anti-CD9, anti- CD63, and anti-β-actin were obtained from Santa Cruz.

### Dual-luciferase reporter gene assay

The 3′-UTR fragments of wild-type/mutated NFIA or LIFR-AS1 were cloned into the pmirGLO luciferase reporter vector. Then, they were co-transfected into 293 T cells with miR-29a mimic/NC. Dual-Luciferase Reporter Assay System (E191, Promega) was used to detect the luciferase activity and the results are expressed as firefly luciferase activity normalized to Renilla luciferase activity.

### Flow cytometry (FCM)

For apoptosis, cells were collected after 24 h of culture and stained with the Annexin V/FITC Kit (BD Biosciences) according to the guidelines provided. And then, flow cytometry was used to analyze apoptosis.

### Wound-healing and transwell assay

For the wound-healing assay, cells were added in 6-well plates and cultured in an incubator. When the confluency reached 70–80%, artificial wounds were made by P-200 pipette tips. The images of 0 h and 24 h were acquired under a phase-contrast microscope.

For transwell assay, 600 μl complete medium was added into the lower chambers of transwell plates (BD Biosciences). And then 5 × 10^4^ cells in 200 μl FBS-free medium were seeded into the matrigel-coated or non-coated upper chambers. After 24 h, the medium was discarded and cells were fixed with 4% paraformaldehyde for 20 min, then washed with PBS and stained with 0.1% crystalline violet solution for analyzing under a microscope.

### Exosomes extraction and exosome uptake assay

Cells were incubated in an exosome-free medium for 48 h and the culture supernatants were collected to centrifuged as following steps: 300×*g* for 10 min to remove cells, 2000×*g* for 10 min to remove dead cells, 10,000×*g* for 30 min to remove cell debris, 100,000×*g* for 70 min to collect pellets and washed with PBS, 100,000×*g* for 70 min to collect exosomes.

PKH26 Fluorescent Cell Linker Kits (Sigma) was used to label macrophage-derived exosomes according to the manufacturer’s instructions. Then, PKH26-labeled exosomes were added into osteosarcoma cells and the uptake process was recorded by a confocal fluorescence microscope.

### Cck-8, colony formation and EdU assay

For the cck-8 assay, 5 × 10^3^ cells were seeded into 96-well plates. And cell viability was examined daily for 3 days using CCK-8 (Dojindo Laboratories, Japan) according to the instruction provided.

For colony formation assay, 800 cells were seeded into 12-well plates and cultured for 4–5 days. Then, the medium was discarded and cells were fixed with 4% paraformaldehyde for 20 min, washed with PBS, and stained with 0.1% crystalline violet solution for analysis.

For EdU assays, 5 × 10^3^ cells were seeded into 96-well plates and EdU Apollo 567 Cell Tracking Kit (RiboBio, China) was used according to the instruction provided. And cell nucleus was stained with DAPI.

### Tumor xenografts

22 BALB/c nude mice were purchased from Vital River (Beijing, China). For tumor xenograft models, 5 × 10^6^ 143B cells were subcutaneously injected in the right flank of 6 mice, and after 3 days, THP-1 induced macrophage-derived exosomes (1 mg/kg in 100 μl PBS) or PBS was locally injected into the tumor mass once every 3 days for 5 times. The tumor volume (length × width^2^/2) was recorded every 3 days and the mice were sacrificed after 15 days. Furthermore, we repeated the animal experiment using peripheral blood mononuclear cells (PBMCs) derived macrophage in another 10 mice (5 mice per group).

For lung metastasis models, 2 × 10^6^ 143B cells were intravenously injected in the tail vein of 6 mice. Then, macrophages-derived exosomes (1 mg/kg in 100 μl PBS) or PBS were injected into the tail vein of mice once every 3 days for 5 times. The mice were sacrificed after 15 days and lungs were collected for further research.

All animal experiments were performed with written confirmation authorized by the Animal Care and Use Committee of Peking University People’s Hospital. Animal experiments complied with the ARRIVE guidelines and followed the National Institutes of Health Guide for the Care and Use of Laboratory Animals.

### Statistical analysis

SPSS 21.0 software was used for statistical analyses and data were presented as the mean ± SD. Differences between groups were analyzed by Student’s t-test.

## Result

### Macrophages promote the proliferation and invasion but inhibit apoptosis of osteosarcoma cells

**Fig. 1:**
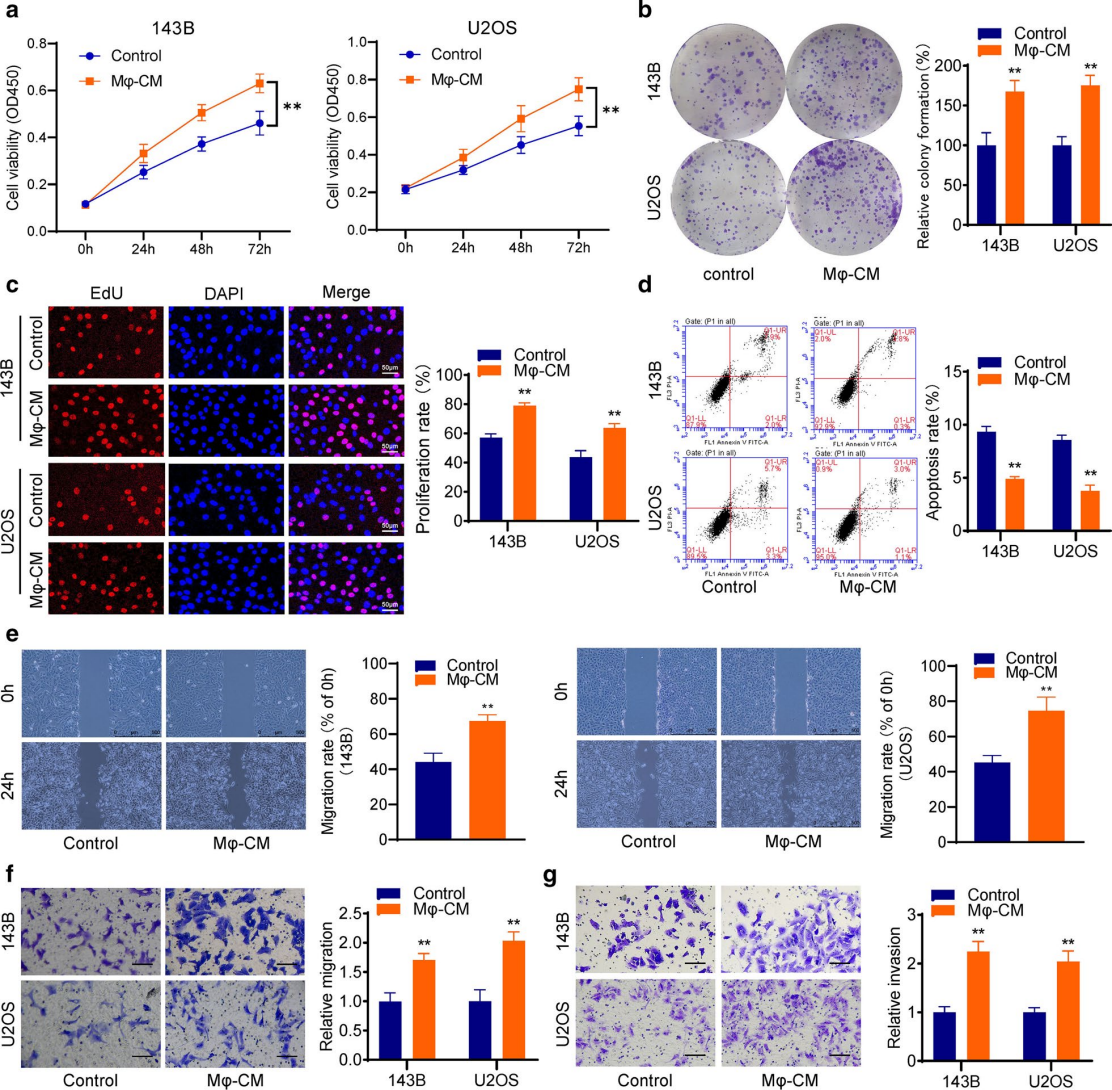
Macrophages promote the proliferation and invasion but inhibit apoptosis of osteosarcoma cells. **a** The cck-8 assay for cell proliferation. **b** Colony formation assay. **c** EdU assay for cell proliferation. **d** Flow cytometry was used to detect the apoptosis of osteosarcoma cells. **e** Wound-healing assay results showed Mφ-CM could promote the migration of 143B and U2OS cells. **f**–**g** Transwell assays indicated that macrophages could promote both migration and invasion of osteosarcoma cells. **p < 0.01

After THP-1-induced macrophages were incubated in a serum-free medium for 24 h, the culture supernatants were collected as conditioned medium (Mφ-CM). Then, osteosarcoma cells were co-cultured with CM, and functional assays were performed to investigate the effects of macrophages on osteosarcoma cells. The cck-8 assay results showed that Mφ-CM significantly facilitated the proliferation of osteosarcoma cell lines 143B and U2OS (Fig. 1a), and the colony formation assay (Fig. 1b) and EdU assay (Fig. 1c) showed the same results. However, flow cytometry results showed that Mφ-CM significantly inhibited the apoptosis of osteosarcoma cells (Fig. 1d), which indicated Mφ-CM might promote cell proliferation by restrain apoptosis in osteosarcoma. Besides, wound-healing assay results showed Mφ-CM could promote the migration of 143B and U2OS cells (Fig. 1e). And then, the transwell assays verified that both migration ability and invasion ability of osteosarcoma cells were increased by Mφ-CM (Fig. 1f–g). Then, we verified the results using peripheral blood mononuclear cells (PBMCs) derived macrophage, which showed the same tumor-promoting action as THP-1 induced macrophages (Additional file 1: Figure S1).

### LncRNA LIFR-AS1 is highly expressed in Mφ-CM co-cultured osteosarcoma cells and acts as a sponge for miR-29a

**Fig. 2:**
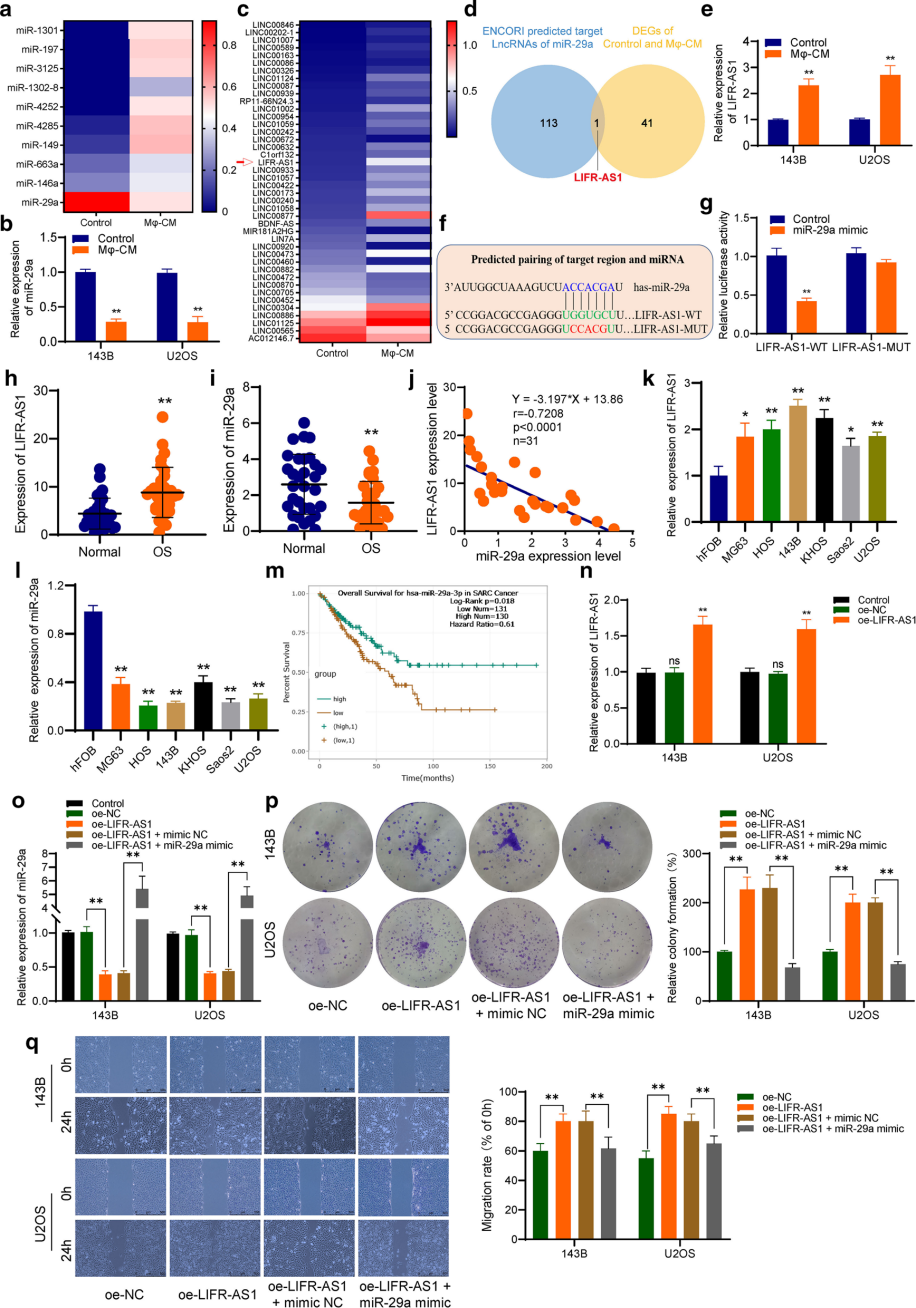
LncRNA LIFR-AS1 is highly expressed in Mφ-CM co-cultured osteosarcoma cells and acts as a sponge for miR-29a. **a** Heatmap showing the differentially expressed miRNAs. **b** the expression of miR-29a was verified using qRT-PCR. **c** Heatmap representing the differentially expressed lncRNAs. **d** LIFR-AS1 was the only intersecting gene between predicted target lncRNAs of miR-29a and differentially expressed lncRNAs. **e** The expression of LIFR-AS1 was verified using qRT-PCR. **f** Schematic representation of the binding site between LIFR-AS1 and miR-29a. **g** The dual-luciferase reporter assay verified the direct binding between LIFR-AS1 and miR-29a. **h**–**j** LIFR-AS1 was highly expressed and miR-29a was lowly expressed in osteosarcoma tissues, and negatively correlated.** k**–**l** The expression of LIFR-AS1 and miR-29a was detected in osteosarcoma cell lines and hFOB. **m** ENCORI database revealed that there was a significant positive correlation between miR-29a expression and overall survival. **n–o** The expression of LIFR-AS1 and miR-29a in osteosarcoma cells. **p**–**q** Overexpressed LIFR-AS1 can significantly promote proliferation and invasion of osteosarcoma cells, but miR-29a overexpression can rescue its effects. oe-LIFR-AS1: LIFR-AS1 overexpressed; oe-LIFR-AS1 + mimic NC: co-transfecting LIFR-AS1 overexpressed plasmid and miRNA mimics NC; oe-LIFR-AS1 + miR-29a mimic: co-transfecting LIFR-AS1 overexpressed plasmid and miR-29a mimic. *p < 0.05, **p < 0.01

RNA-sequencing was used to identify the differentially expressed miRNAs and lncRNAs in Mφ-CM co-cultured osteosarcoma cells and the corresponding control group. As showed in Fig. 2a–b, lowly expressed miR-29a was observed in Mφ-CM co-cultured osteosarcoma cells compared with the control group. Then, the ENCORI database was used to predict target lncRNAs of miR-29a, and highly expressed LIFR-AS1 was the only intersecting gene between predicted target lncRNAs and differentially expressed lncRNAs (Fig. 2c–e). To investigate the interaction between LIFR-AS1 and miR-29a, a dual-luciferase reporter assay was performed and the results showed that miR-29a mimic significantly reduced luciferase activity in LIFR-AS1 wild type (WT), but not mutant (MUT), verifying the direct binding between LIFR-AS1 and miR-29a (Fig. 2f–g). Furthermore, we found that lncRNA LIFR-AS1 was highly expressed and miR-29a was lowly expressed in osteosarcoma tissues compared with normal tissues, and there is a negative correlation between their expression (Fig. 2h–j). The same result was observed in osteosarcoma cell lines when compared with human osteoblast cell line hFOB (Fig. 2k–l). Besides, the ENCORI database revealed that there was a significant positive correlation between miR-29a expression and overall survival (p = 0.018) (Fig. 2m).

To further confirm the function of LIFR-AS1 in tumorigenesis. We directly overexpressed lncRNA LIFR-AS1 in osteosarcoma cells and found that overexpressed LIFR-AS1 can significantly promote proliferation and invasion of osteosarcoma cells. Furthermore, miR-29a overexpression can rescue the effects of LIFR-AS1 overexpression (Fig. 2n–q).

### LIFR-AS1 can be transmitted from macrophages to osteosarcoma cells via exosomes

**Fig. 3:**
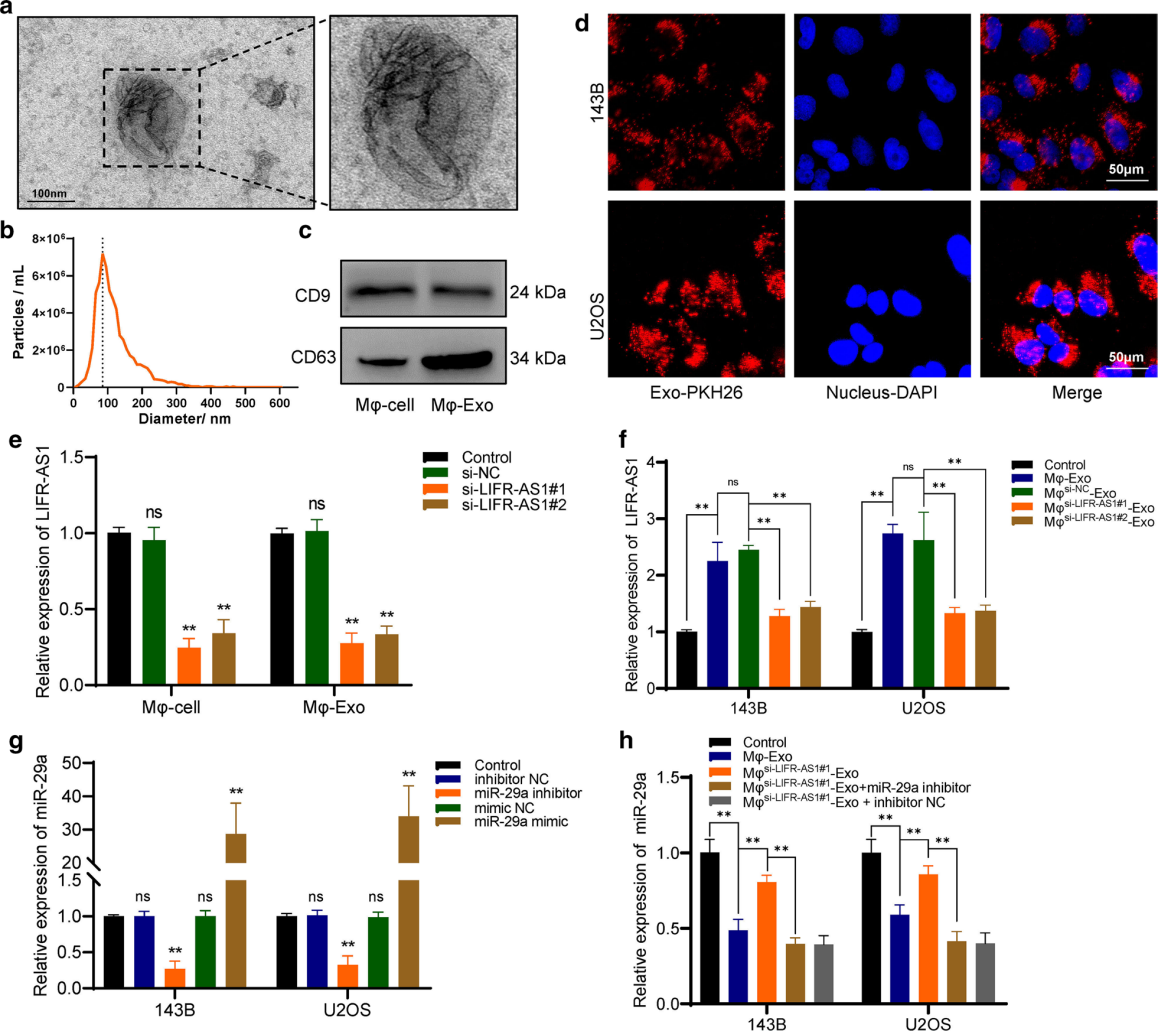
LIFR-AS1 can be transmitted from macrophages to osteosarcoma cells via exosomes. **a** Representative image of exosomes under TEM. **b** NTA results showed that Mφ-Exos were located predominately around 100 nm. **c** Exosome surface marker proteins CD9, CD63 was detected by western blot. **d** Macrophages-derived exosomes were ingested by 143B cells. **e** LIFR-AS1 was knocked down by siRNAs in both macrophages and Mφ-Exos. **f** The expression of LIFR-AS1 in Mφ-Exos co-cultured osteosarcoma cells. **g** The expression of miR-29a can be regulated by miR-29a mimics and inhibitors in osteosarcoma cells. **h** The expression of miR-29a was detected under different conditions. Mφ^si−LIFR−AS1#1^-Exo + miR-29a inhibitor/inhibitor NC: Cells were transfected with miR-29a inhibitor/inhibitor NC and co-cultured with LIFR-AS1- knockdown Mφ-Exos. *p < 0.05, ns = no significant

macrophages-derived exosomes (Mφ-Exos) were extracted using ultracentrifugation, and the exosome identification was performed by transmission electron microscopy (TEM), Nanoparticle Tracking Analysis (NTA), and western blot. Under TEM, Mφ-Exos were observed as a bowl-shaped bilayer membrane structure with diameters range of around 100 nm (Fig. 3a). NTA results showed that Mφ-Exos were located predominately around 100 nm, which was consistent with the TEM result (Fig. 3b). Western blot results confirmed that exosome surface marker proteins CD9 and CD63 could be detected in Mφ-Exos (Fig. 3c). Then, an exosome uptake assay was performed and the transport processes of exosomes could be observed under a confocal fluorescence microscope (Fig. 3d). Furthermore, two siRNA sequences were used to knock down the expression of LIFR-AS1 in macrophages, and the results showed that LIFR-AS1 was significantly downregulated by siRNAs in both macrophages and Mφ-Exos (Fig. 3e), and the same results were observed in Mφ-Exos co-cultured osteosarcoma cells (Fig. 3f). Besides, after co-cultured for 24 h, qRT-PCR results showed that miR-29a was significantly downregulated by Mφ-Exos in osteosarcomas, and this effect was attenuated when LIFR-AS1 was knocked down in Mφ-Exos. And, miR-29a inhibitor could significantly downregulate the expression of miR-29a in both osteosarcoma cells and Mφ-Exos co-cultured osteosarcoma cells (Fig. 3g–h). We further detected the expression of miR-29a in both macrophage cells and exosomes after lncRNA LIFR-AS knockdown in macrophages (Additional file 1: Figure S3). And the result showed that LIFR-AS knockdown can significantly upregulated the expression of miR-29a in macrophages cells but not in exosomes, which could be attributed to the selective encapsulation of exosomes.

### Macrophages-derived exosomal LIFR-AS1 promotes osteosarcoma cell progression via sponging miR-29a

**Fig. 4:**
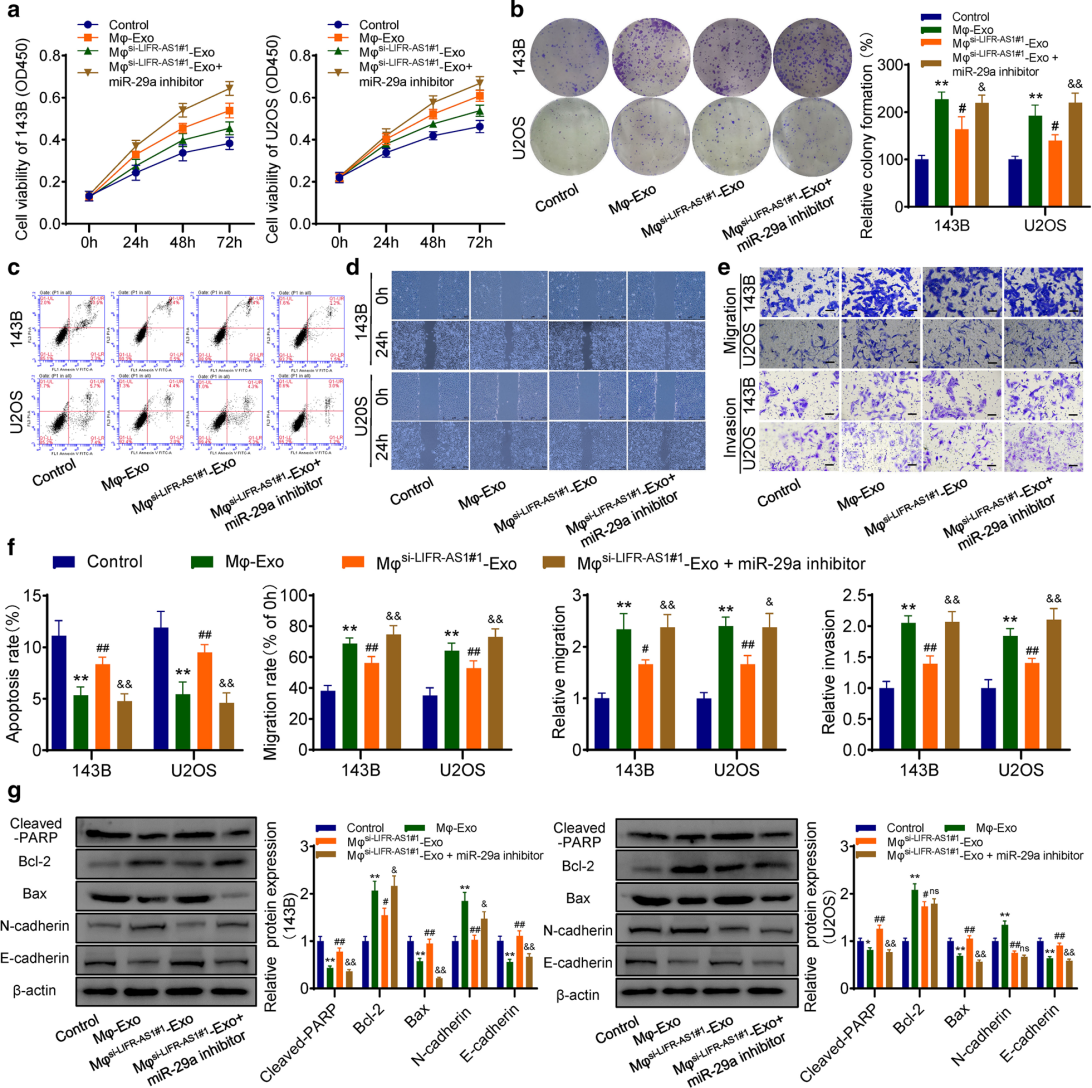
Macrophages-derived exosomal LIFR-AS1 promotes osteosarcoma cell progression via sponging miR-29a. **a** The cck-8 assay for cell proliferation. **b** Colony formation assay. **c** Flow cytometry for apoptosis. **d** Wound-healing assay. **e** Transwell assays for migration and invasion. **f** Statistical results of apoptosis, wound-healing, and transwell assays are showing accordingly. **g** The expression levels of apoptosis-related and EMT-related proteins were detected by western blot. Mφ^si−LIFR−AS1#1^-Exo + miR-29a inhibitor: Cells were transfected with miR-29a inhibitor/inhibitor NC and co-cultured with LIFR-AS1- knockdown Mφ-Exos. *p < 0.05, **p < 0.01, compared with control group; #p < 0.05, ##p < 0.01, compared with Mφ-Exo group; &p < 0.05, &&p < 0.01, compared with Mφ^si−LIFR−AS1#1^-Exo group

Both cck-8 and colony formation assays results showed that Mφ-Exos significantly promoted osteosarcoma cell proliferation, and the promoting effect was attenuated when LIFR-AS1 was knocked down in Mφ-Exos (Mφ^si−LIFR−AS1^-Exo). However, miR-29a inhibitor could partially reverse the effect of Mφ^si−LIFR−AS1^-Exo on osteosarcoma cells (Fig. 4a–b). Flow cytometry results showed that Mφ-Exos suppressed osteosarcoma cells apoptosis, and miR-29a inhibitor could partially recover LIFR-AS1-knockdown caused effect on osteosarcoma cells (Fig. 4c). Similarly, the results of the wound-healing assay and transwell assay demonstrated that Mφ-Exos significantly enhanced the migration and invasion abilities of osteosarcoma cells, and miR-29a inhibitor had the opposite effect with macrophages-derived exosomal LIFR-AS1 (Fig. 4d–f). Furthermore, we detected the apoptosis and epithelial-mesenchymal transition (EMT) associated proteins in osteosarcoma cells via western blot, and the results showed that Mφ-Exos significantly inhibited the expression of apoptosis protein cleaved-PARP, Bax, and EMT marker E-cadherin, but promoted the expression of anti-apoptosis protein Bcl-2, and EMT marker N-cadherin. However, exosomal LIFR-AS1-knockdown attenuated the regulating effect of Mφ-Exos on osteosarcoma cells, and miR-29a inhibitor could partially recover it (Fig. 4g). Taken together, all these results indicated that macrophages could promote osteosarcoma cell progression via exosomal LIFR-AS1, which could act as a sponge for miR-29a in osteosarcoma cells.

### NFIA is the direct target gene of miR-29a

**Fig. 5:**
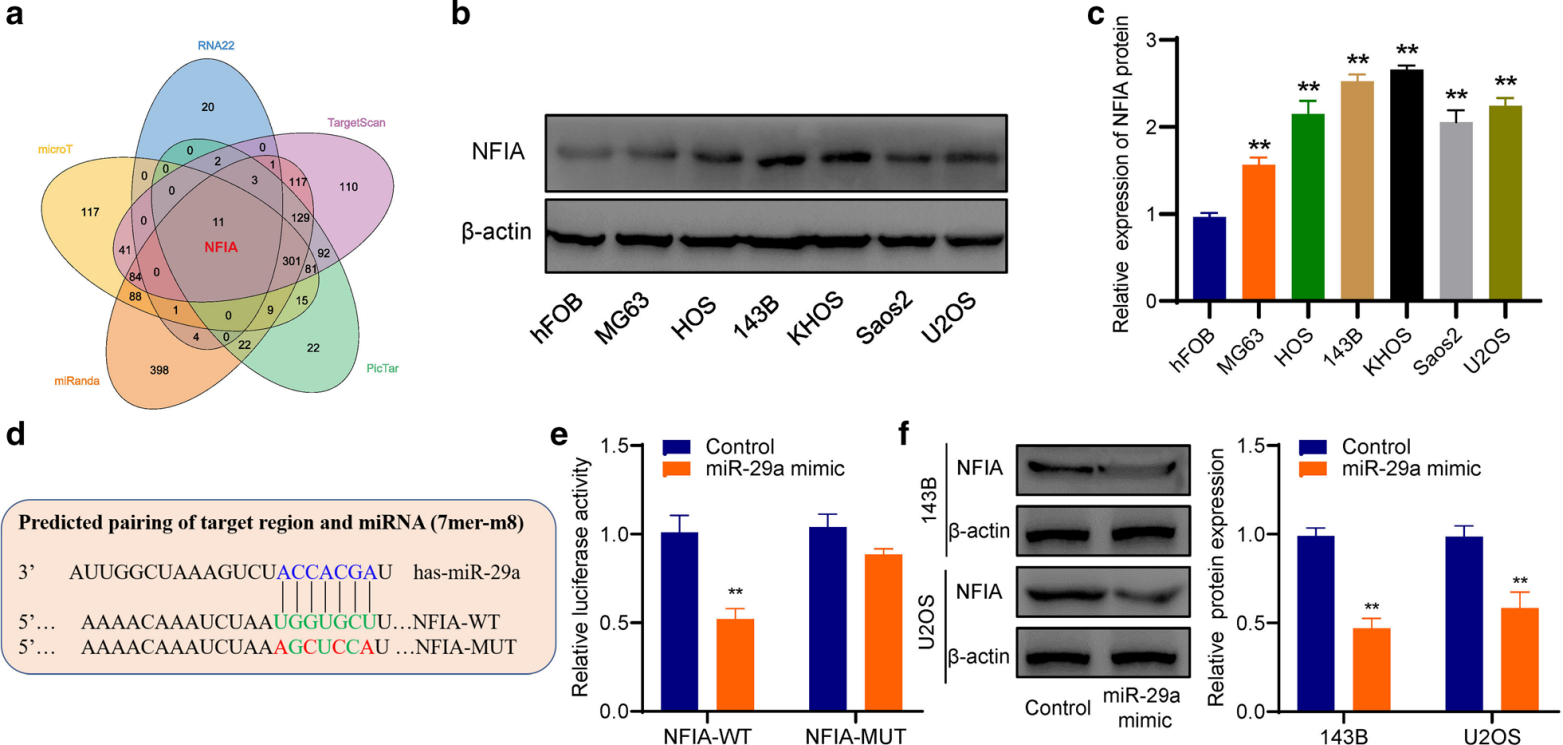
NFIA is the direct target gene of miR-29a. **a** Venn diagram of the predicted targeting genes of miR-29a. **b**–**c** The expression of NFIA protein was detected in osteosarcoma cell lines and human osteoblast cell Line hFOB. **d** schematic representation of NFIA 3′-UTR containing wild-type or mutant binding site for miR-29a. **e** The dual-luciferase reporter assay verified the direct binding between NFIA and miR-29a. **f** Western blot confirmed that NFIA protein was downregulated by miR-29a mimics in osteosarcoma cells. **p < 0.01

Five bioinformatics tools (RNA22, micro T, TargetScan, PicTar, and miRanda) were used to predict the target gene of miR-29a, and nuclear factor I/A (NFIA) was identified as a possible target gene according to the prediction score and publications (Fig. 5a). Then, the expression of NFIA protein was detected by western blot, and the result indicated that NFIA was highly expressed in osteosarcoma cell lines (143B, HOS, MG63, MNNG, Saos2 and U2OS) compared with human osteoblast cell Line hFOB (Fig. 5b–c). Furthermore, the direct binding between miR-29a and NFIA was verified by dual-luciferase reporter assay (Fig. 5d–e). And the result of western blot confirmed that NFIA protein expression could be regulated by miR-29a (Fig. 5f).

### miR-29a inhibited the proliferation and invasion but induced apoptosis of osteosarcoma cells via targeting NFIA

**Fig. 6:**
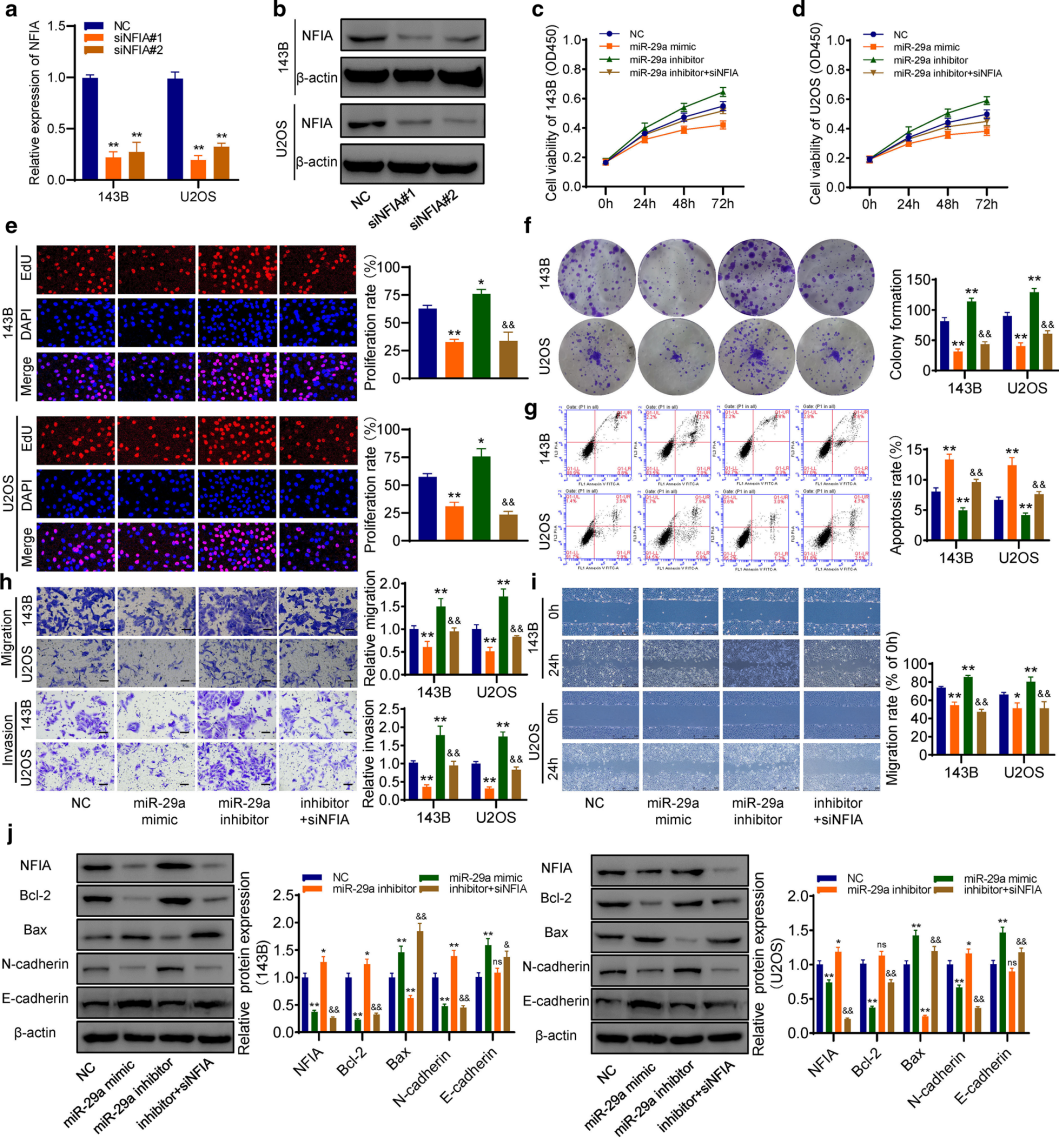
miR-29a inhibited the proliferation and invasion, but induced apoptosis of osteosarcoma cells via targeting NFIA. **a**–**b** NFIA was knocked down in both mRNA and protein levels. **c**–**d** The cck-8 assay. **e** Colony formation assay. **f** The EdU assay for proliferation. **g** Flow cytometry for apoptosis. **h** The transwell assay for migration and invasion. **i** The wound-healing assay. **j** The expression levels of NFIA, apoptosis-related, and EMT-related proteins were detected by western blot. Inhibitor + siNFIA: co-transfecting miR-29a inhibitor and siNFIA. *p < 0.05, **p < 0.01, compared with control group; &p < 0.05, &&p < 0.01, compared with miR-29a inhibitor group. ns = no significant

Two siRNA sequences were transfected into osteosarcoma cells, and the knockdown effect was observed in both mRNA and protein levels (Fig. 6a–b). The results of cck-8 (Fig. 6c–d), colony formation (Fig. 6e) and EdU assays (Fig. 6f) showed that miR-29a significantly inhibited the proliferation of osteosarcoma cells, and NFIA-knockdown could partially reverse miR-29a inhibitor-mediated cell proliferation promotion. Flow cytometry results showed that miR-29a could induce apoptosis of osteosarcoma cells, and NFIA-knockdown could recover miR-29a inhibitor-mediated apoptosis inhibition (Fig. 6g). Besides, the results of the wound-healing assay and transwell assay demonstrated that miR-29a significantly inhibited the migration and invasion abilities of osteosarcoma cells, and NFIA-knockdown could recover miR-29a inhibitor-mediated cell migration promotion and invasion promotion (Fig. 6h and i). Furthermore, western blot results showed that miR-29a significantly inhibited the expression of NFIA, anti-apoptosis protein Bcl-2, and EMT marker N-cadherin, but promoted the expression of pro-apoptotic protein Bax and EMT marker E-cadherin in osteosarcoma cells. However, miR-29a inhibitor showed opposite regulation on these proteins and could be reversed by NFIA-knockdown (Fig. 6j).

### NFIA is highly expressed in pulmonary metastasis focuses of osteosarcoma patient

**Fig. 7:**
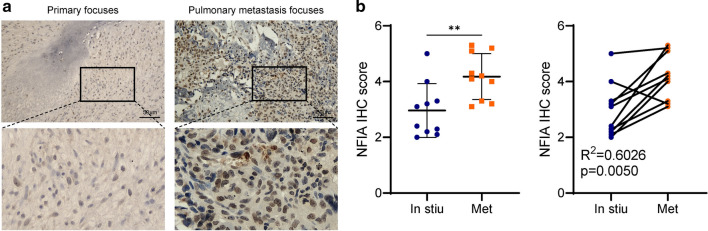
NFIA is highly expressed in pulmonary metastasis focuses of osteosarcoma patient. **a** Representative image of IHC result. **b** IHC result showed that NFIA was expressed more strongly in pulmonary metastasis focuses. **p < 0.01

The expression of NFIA was examined in human primary osteosarcoma tissue and corresponding lung metastases (n = 10 pairs), and the result showed that NFIA was expressed more strongly in pulmonary metastasis focuses (Fig. 7a–b).

### Macrophages-derived exosomes promote osteosarcoma growth and metastasis in vivo

**Fig. 8:**
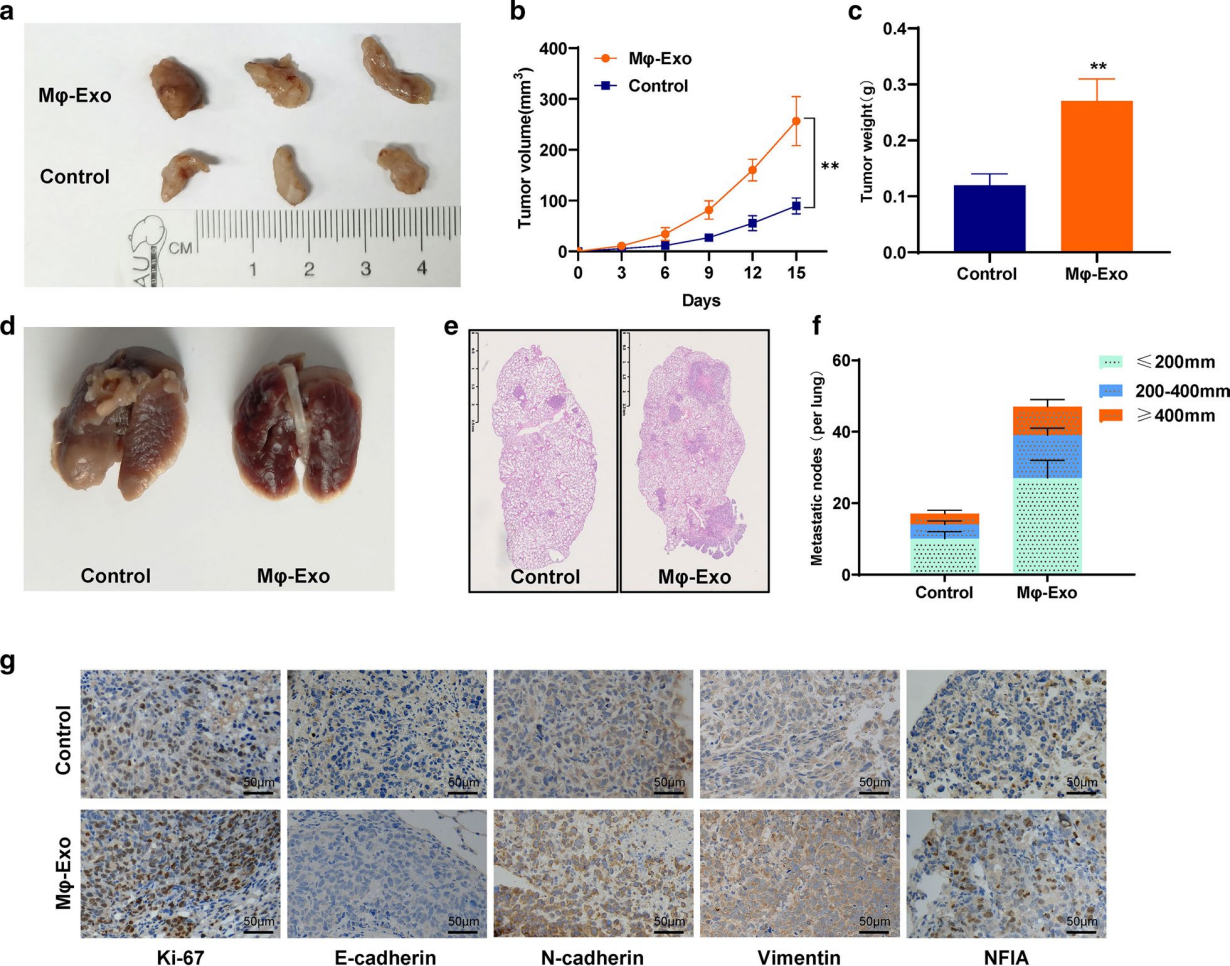
Macrophages-derived exosomes promote osteosarcoma growth and metastasis in vivo. **a** The effect of Mφ-Exos on the growth of tumors was detected on xenograft. **b** The growth curve of xenograft. **c** The tumor weight of xenograft. **d** The lung metastasis model was established via a tail vein injection of osteosarcoma cells. **e**–**f** Metastatic tumor nodules were observed by H&E staining. **g** IHC was performed to detect the expression of proteins. **p < 0.01

The effect of Mφ-Exos on the growth of tumors was detected on xenograft in nude mice, and the results indicated that Mφ-Exos significantly promoted the growth rate and increased the tumor weight of osteosarcoma (Fig. 8a–c, and Additional file 1: Figure S2). Then, the lung metastasis model was established via a tail vein injection of osteosarcoma cells, and more metastatic tumor nodules were observed in the Mφ-Exos group compared with the control group (Fig. 8d–f). Furthermore, IHC was performed to detect the expression of related proteins, and the result indicated that the tumors of the Mφ-Exos group expressed more Ki-67, N-cadherin, Vimentin, and NFIA, but less E-cadherin than those of the control group (Fig. 8g).

## Discussion

Osteosarcoma patients often have a poor prognosis due to the rapid progression and lack of effective therapy [19]. Macrophages, as the most abundant infiltrating immune cells in the tumor microenvironment, play a pivotal role in tumorigenesis, tumor development, and tumor therapy [20]. Recently, more and more studies indicate that macrophages invade massively osteosarcoma tissues and appear to play a crucial role in the development of osteosarcoma [21]. However, due to the infiltration and polarization of macrophages are dynamically changed in tumors, the function of macrophages in osteosarcoma is also inconsistent [11]. Buddingh et al. demonstrated that the total number of tumor-infiltrating macrophages was related to metastasis suppression but M2 macrophages were associated with poor prognosis in high-grade osteosarcoma [22]. Zhou et al. reported that M2 macrophages promote pulmonary metastasis of osteosarcoma [23]. And our previous study revealed that macrophages contribute to metastasis and invasion in osteosarcoma patients [11].

On the other hand, the specific communication mechanism between macrophages and osteosarcoma cells is still largely unclear. Su et al. reported that macrophage-derived CCL18 promotes osteosarcoma proliferation and migration by upregulating the expression of UCA1 [24]. In the previous study, we found that macrophages CM could promote the progression of osteosarcoma cells [11]. And in this study, we further confirm that Mφ-CM can regulate several processes of osteosarcoma, including proliferation, invasion, migration, and apoptosis etc.

To further explore the specific molecular mechanisms, in this report, we use RNA-sequencing to identify the differentially expressed miRNAs and lncRNAs in Mφ-CM co-cultured osteosarcoma cells and the corresponding control group, and lowly expressed miR-29a is observed in Mφ-CM co-cultured osteosarcoma cells. Furthermore, we confirm that lncRNA LIFR-AS1 is upregulated in Mφ-CM co-cultured osteosarcoma cells and can act as a sponge of miR-29a. Emerging studies have suggested that exosomes can act as a crucial mediator between cellular communication by transporting cargos like lncRNAs [25, 26]. In this study, we also demonstrate that LIFR-AS1 can be transmitted from macrophages to osteosarcoma cells via exosomes and further promote the tumor progression via sponging miR-29a.

LIFR-AS1 is a newly described tumor-related lncRNA and can serve as a competitive endogenous RNA (ceRNA) for several miRNAs. Wang et al. reported that LIFR-AS1 was lowly expressed in breast cancer and associated with poor prognosis [27]. Zhu et al. revealed that LIFR-AS1 could be a potential therapeutic target as well as a prognostic biomarker of clear cell kidney carcinoma [28]. Also, Liu et al. reported that LIFR-AS1 modulates the resistance of colorectal cancer to photodynamic therapy via the miR-29a/ TNFAIP3 axis [29].

Besides, lots of studies have demonstrated that dysregulated miRNAs were vital regulators in the development of various tumors [18]. And miR-29a is a widely researched miRNA, it can act as either oncogene or tumor suppressor in a variety of tumors [30]. In the present study, we find that miR-29a is lowly expressed in osteosarcoma tissues compared with normal tissues, and it can inhibit the progression of osteosarcoma cells via targeting NFIA. Consistently, Gong et al. reported that lowly expressed miR-29a inhibited the invasion and migration of osteosarcoma cells via targeting DNMT3B [31]. Liu et al. reported that miR-29a could inhibit adhesion, migration, and invasion of osteosarcoma cells by suppressing CDC42 [32].

NFIA is a member of the nuclear factor I (NFI) family and plays an essential role in tumors. Overexpression of NFIA has been reported in esophageal squamous carcinoma and Glioblastomas and plays a tumor-promoting role [33, 34]. In this study, NFIA is predicted as a target gene of miR-29a via several databases and then it is validated by further researches.

**Fig. 9:**
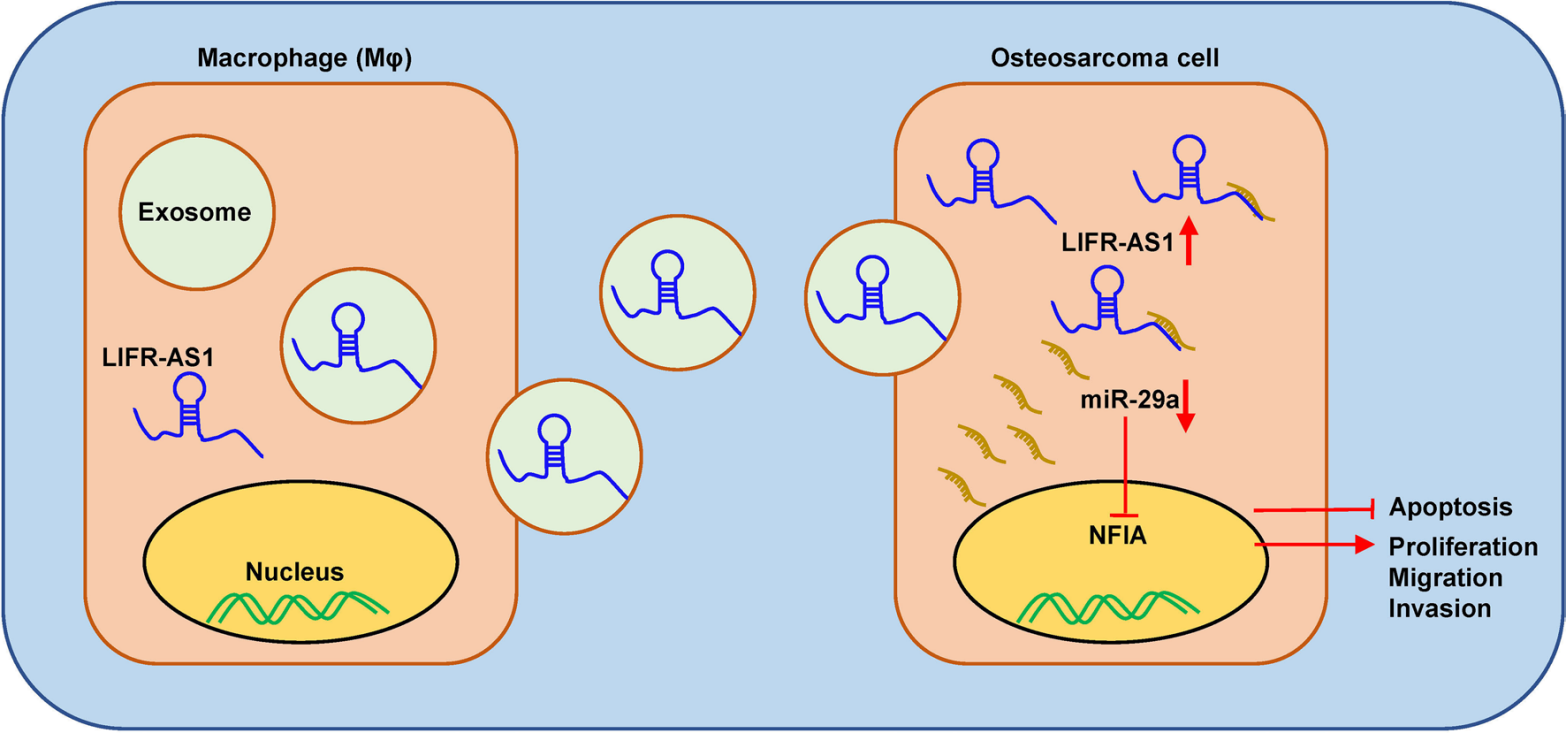
A schematic drawing of macrophages-derived exosomal LIFR-AS1 enter osteosarcoma cells. LncRNA LIFR-AS1 is loaded in macrophages-derived exosomes and then delivered to osteosarcoma cells. In osteosarcoma cells, increased LncRNA LIFR-AS1 promoted proliferation, migration, and invasion of tumor cells and inhibit their apoptosis via miR-29a/NFIA/Smad3 axis

In conclusion, our results revealed that macrophages-derived exosomes played an important role in osteosarcoma development and macrophages-derived exosomal lncRNA LIFR-AS1 promoted osteosarcoma cell proliferation and invasion via miR-29a/NFIA axis (Fig. 9). This study provided a new insight into the interaction between osteosarcoma cells and macrophages in the tumor microenvironment, and highlight the potential of LIFR-AS1/miR-29a/NFIA as novel therapeutic targets for osteosarcoma therapy.

## Supplementary Information

**Additional file 1: Figure S1.** Peripheral blood mononuclear cells (PBMCs) derived macrophage promote the proliferation and invasion of osteosarcoma cells. (**A**) The cck-8 assay for cell proliferation. (**B**) Colony formation assay. (**C-D**) Wound-healing assay. **p < 0.01. **Figure S2.** PBMCs induced Macrophages-derived exosomes promote osteosarcoma growth in vivo. (**A**) The effect of Mφ(PBMC)-Exos on the growth of tumors was detected on xenograft. (**B**) The growth curve of xenograft. **p < 0.01. **Figure S3.** The expression of miR-29a in both macrophage cells and exosomes after lncRNA LIFR-AS knockdown in macrophages. The result showed that LIFR-AS knockdown can significantly upregulated the expression of miR-29a in macrophages cells but not in exosomes. **p < 0.01, ns=no significant.

## Data Availability

The datasets used and/or analyzed during the current study are available from the corresponding author on reasonable request.

## Abbreviations

TAMs: Tumor-associated macrophages
qRT-PCR: Quantitative real-time polymerase chain reaction
LIFR-AS1: LIFR antisense RNA 1
OS: Osteosarcoma
lncRNA: Long non-coding RNA
NFIA: Nuclear factor I A
FCM: Flow cytometry
Cck-8: Cell counting kit-8
EdU: 5-Ethynyl-2′-deoxyuridine
PBS: Phosphate buffer saline
