# Supplementary figures and images for: Macrophages-derived exosomal lncRNA LIFR-AS1 promotes osteosarcoma cell progression via miR-29a/NFIA axis
Source: Cancer Cell Int. 2021 Apr 1;21:192. doi: 10.1186/s12935-021-01893-0 (PMC8017664; doi:10.1186/s12935-021-01893-0)

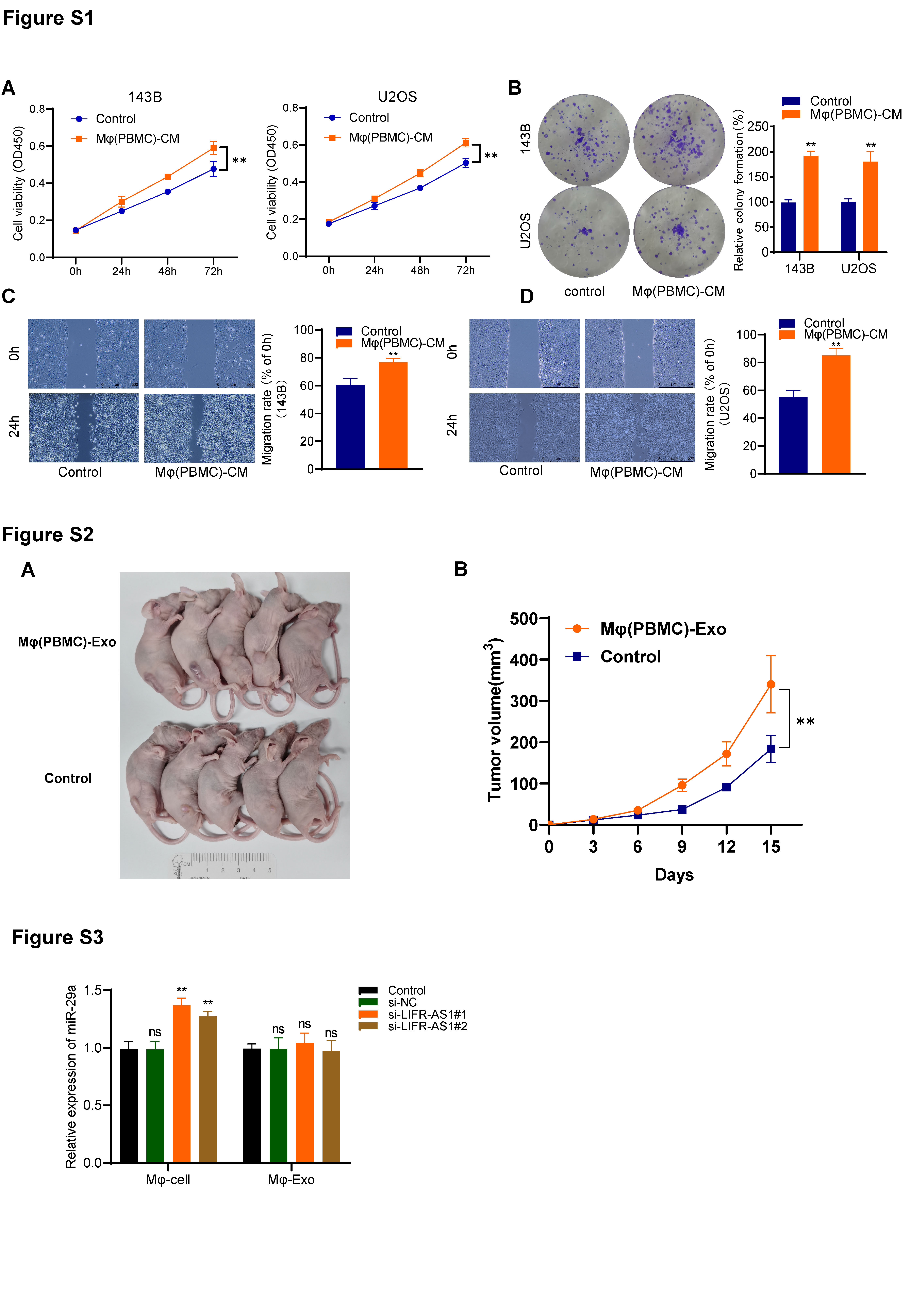

Supplement: Supplementary file 1 — Additional file 1: Figure S1. Peripheral blood mononuclear cells (PBMCs) derived macrophage promote the proliferation and invasion of osteosarcoma cells. (A) The cck-8 assay for cell proliferation. (B) Colony formation assay. (C-D) Wound-healing assay. **p < 0.01. Figure S2. PBMCs induced Macrophages-derived exosomes promote osteosarcoma growth in vivo. (A) The effect of Mφ(PBMC)-Exos on the growth of tumors was detected on xenograft. (B) The growth curve of xenograft. **p < 0.01. Figure S3. The expression of miR-29a in both macrophage cells and exosomes after lncRNA LIFR-AS knockdown in macrophages. The result showed that LIFR-AS knockdown can significantly upregulated the expression of miR-29a in macrophages cells but not in exosomes. **p < 0.01, ns=no significant. [file 12935_2021_1893_MOESM1_ESM.jpg]
